# 

**DOI:** 10.1017/psy.2025.10077

**Published:** 2026-01-27

**Authors:** Yustus Dwi Putera Sepverson Babys

**Affiliations:** Psychology, Airlangga University Faculty of Psychology, Indonesia

Psychological assessment is fundamental to understanding human cognition, emotion, and behavior. Yet, both traditional and contemporary approaches continue to face challenges including measurement bias, cultural variation, and the growing digitization of test delivery that affect fairness, validity, and reliability. These issues underscore the importance of employing rigorous psychometric principles and advanced statistical tools to ensure assessments remain credible and meaningful.

Several empirical studies highlight these challenges. Macqueen et al. (2024) examined psychological testing practices among Australian professionals and identified diverse attitudes toward online assessments, low rates of instrument adaptation, limited generalizability, and psychometric limitations in the measures employed. Sam (2024) called for re-evaluating the objectives of acculturation research in light of societal demographic change and recommended the use of advanced statistical methods to sustain its scientific contribution. Lee and Kim (2022) analyzed how digital platforms influence test fairness, while Johnson et al. (2021) discussed adapting legacy instruments for digital contexts.

*Principles of Psychological Assessment with Applied Examples in R* advocates for empirical rigor in evaluating assessment instruments and illustrates how modern psychometric frameworks and R-based workflows can enhance validity, reliability, and generalizability. The book also addresses ethical and cultural considerations in assessment practice and integrates open-science principles with practical R applications.

Adopting an epistemological orientation, the author clarifies systematic approaches to producing and validating measurement evidence. Its dual focus on conceptual foundations and executable R examples makes it a valuable resource for readers with intermediate quantitative skills and familiarity with research design.

The book is organized into 24 chapters that combine conceptual discussion with R code demonstrations. Chapters 1–4 introduce core concepts such as score types, scale selection, and psychometric properties of reliability and validity. Chapters 5–8 expand to generalizability theory, factor analysis, structural equation modeling, and item response theory, offering guidelines to avoid common misapplications. Chapters 9–12 address predictive approaches, ethics, and evidence-based instrument selection. Later chapters illustrate personality, cognitive, and behavioral assessments as well as adaptive testing and cross-cultural considerations. This progression from foundational to applied topics provides a clear narrative structure for readers to follow.

A central strength of the book lies in its integration of R code and reproducible examples, enabling readers to replicate analytical steps and customize workflows for their own datasets. This feature enhances accessibility for practitioners and supports the adoption of transparent psychometric practices.

Nevertheless, some chapters prioritize technical illustrations over extended theoretical discussion. Incorporating explicit case studies and ethical scenarios would further strengthen its practical value. The R-based focus may challenge novice readers, but the inclusion of step-by-step tutorials or supplementary material could help mitigate this limitation. Contemporary guides such as Prokofieva et al. (2023) complement the book by providing structured instruction for complex models like exploratory structural equation modeling.

Chapters 13–16 address the ethical, cultural, and procedural foundations that support fair and responsible psychological assessment. Chapter 13 reviews professional codes of ethics including those of the American Psychological Association (2017) and connects them with the growing open-science movement, emphasizing transparency, informed consent, and the integrity of clinical report writing. Chapter 14 examines intelligence and aptitude testing, discussing how test interpretation can influence educational and clinical decisions. Chapter 15 focuses on measurement bias and differential item functioning, presenting statistical and procedural strategies for detecting and mitigating cultural bias in test design and application. Chapter 16 explores interview-based assessment, comparing the *Diagnostic and Statistical Manual of Mental Disorders* and the International Classification of Diseases frameworks, and critically evaluating their strengths and limitations for diagnostic reliability across cultures.

Chapters 17–20 turn to applied domains of assessment. Chapter 17 reviews objective personality inventories such as the Minnesota Multiphasic Personality Inventory (MMPI), a widely used tool for assessing psychological disorders. The chapter discusses issues related to validity, interpretive accuracy, and clinical utility. Chapter 18 critically evaluates projective techniques including the Thematic Apperception Test and the Rorschach highlighting persistent controversies regarding their empirical foundations and interpretive reliability. Chapter 19 introduces psychophysiological assessment methods, integrating measures such as heart-rate variability and electrodermal activity within clinical and research contexts. Chapter 20 examines computerized adaptive testing, analyzing its psychometric advantaged efficiency, precision, and reduced respondent fatigue while acknowledging technical and fairness challenges associated with algorithmic item selection.

The final section, Chapters 21–24, extends the discussion to behavioral observation, longitudinal design, cognitive assessment, and multicultural applications. Chapter 21 explores behavioral assessment through direct observation and informant reports, stressing inter-rater reliability and ecological validity. Chapter 22 addresses longitudinal and repeated-measures designs, explaining how temporal dynamics influence score interpretation and test selection. Chapter 23 discusses cognitive assessment in relation to anxiety and depression, showing how cognitive testing informs evidence-based interventions such as cognitive-behavioral therapy. The concluding Chapter 24 emphasizes cultural diversity and advocates for multi-method, cross-cultural approaches that combine quantitative precision with contextual sensitivity, thereby enhancing fairness and inclusivity in psychological measurement.

Collectively, these final chapters demonstrate how methodological rigor, ethical responsibility, and cultural awareness converge to strengthen the scientific and practical impact of psychological assessment. They reinforce the book’s overarching message: that robust measurement practices, guided by both statistical sophistication and ethical reflection, are central to advancing contemporary psychometrics.

Although the book does not explicitly define its intended readership, the structure and content suggest it is tailored for graduate students, early-career researchers, and applied practitioners engaged in psychological assessment. The integration of conceptual exposition with executable R code implies a pedagogical orientation toward readers who seek both theoretical grounding and practical analytical skills. Those involved in test development, validation, or clinical interpretation will find the book particularly relevant, as it bridges methodological rigor with real-world application.

To fully benefit from the book’s content, readers are expected to possess foundational knowledge in psychometric theory, statistical modeling, and basic proficiency in the R programming environment. While the author provides illustrative code and workflow examples, the text assumes familiarity with research design and quantitative analysis. Novice readers may require supplementary resources to engage with advanced topics such as structural equation modeling or item response theory. Nonetheless, the book’s clear organization and emphasis on reproducibility offer a supportive framework for learners progressing toward intermediate competence.

In summary, the book offers a coherent, well-edited, and comprehensive overview of modern psychological assessment. It successfully integrates conceptual foundations with reproducible R-based applications, promoting methodological transparency, ethical awareness, and cultural inclusivity. The text stands as a valuable reference for researchers, clinicians, and graduate students who seek to enhance validity, reliability, and fairness in psychological measurement through rigorous empirical methods.

## References

[r1] Johnson, R. , Patel, M. , & Singh, A. (2021). Adapting traditional psychological assessments for the digital age: Challenges and opportunities. Journal of Clinical Psychology, 77(5), 856–874.

[r2] Lee, J. , & Kim, S. (2022). Digital platforms in psychological testing: Impact on fairness and reliability. Journal of Psychological Assessment, 40(2), 112–130.

[r3] Macqueen, P. , Abbott, J. M. , Khawaja, N. G. , Mathews, R. , Scott, D. , & Watt, B. D. (2024). Psychological testing in the profession of psychology: An Australian study. Australian Journal of Psychology, 76(1). 10.1080/00049530.2024.2419682 PMC12218523 40666640

[r4] Prokofieva, M. , Zarate, D. , Parker, A. , Palikara, O. , & Stavropoulos, V. (2023). Exploratory structural equation modeling: A streamlined step-by-step approach using the R project software. BMC Psychiatry, 23(1), Article no. 1. 10.1186/s12888-023-05028-9 PMC10375619 37507658

[r5] Sam, D. L. (2024). Fifty-plus years of psychological acculturation research: Progress and challenges. International Journal of Intercultural Relations, 103, 102076. 10.1016/j.ijintrel.2024.102076

